# The A, B and C’s of Silicone Breast Implants: Anaplastic Large Cell Lymphoma, Biofilm and Capsular Contracture

**DOI:** 10.3390/ma11122393

**Published:** 2018-11-28

**Authors:** Maria Mempin, Honghua Hu, Durdana Chowdhury, Anand Deva, Karen Vickery

**Affiliations:** Faculty of Medicine and Health Sciences, Macquarie University, Macquarie Park, NSW, 2109, Australia; maria.mempin@mq.edu.au (M.M.); helen.hu@mq.edu.au (H.H.); Durdana.chowdhury@hdr.mq.edu.au (D.C.); anand.deva@mq.edu.au (A.D.)

**Keywords:** biofilm, breast implant, textured: capsular contracture, anaplastic large cell lymphoma, BIA-ALCL

## Abstract

Breast implantation either for cosmetic or reconstructive e purposes is one of the most common procedures performed in plastic surgery. Biofilm infection is hypothesised to be involved in the development of both capsular contracture and anaplastic large cell lymphoma (ALCL). Capsular contracture is one of the principal reasons for breast revision surgery and is characterised by the tightening and hardening of the capsule surrounding the implant, and ALCL is an indolent lymphoma found only in women with textured implants. We describe the types of breast implants available with regard to their surface characteristics of surface area and roughness and how this might contribute to capsular contracture and/or biofilm formation. The pathogenesis of capsular contracture is thought to be due to biofilm formation on the implant, which results in on-going inflammation. We describe the current research into breast implant associated ALCL and how implant properties may affect its pathogenesis, with ALCL only occurring in women with textured implants.

## 1. Introduction

Breast implantation, either for cosmetic or reconstructive purposes, is one of the most common procedures performed in plastic surgery. In 2015, in the United States of America alone, more than 280,000 women had breast enlargement surgery and an estimated 106,000 breast cancer patients underwent post-mastectomy breast reconstruction, which often involved insertion of implantable medical devices [1]. 

## 2. Breast Implants

Basic designed silicone breast implants were first introduced in the early 1960s [2]. As each new “generation” of implant has been introduced, their design has undergone major improvements. Modern breast implants can be divided into categories based on implant filling (silicone or saline), surface texture (textured or smooth), and shape (round or anatomic), each of which have slightly different properties [3,4].

Silicone or saline implant filling: 

Saline implants are sold as empty silicone elastomer shells and are filled to the appropriate volume with sterile saline in the operating room. The silicone filling comes as either a “fluid form” that is not cohesive enough to maintain an anatomic shape or a “form-stable” more viscous and greater cross-linked silicone gel that has cohesive properties [5]. The cohesive gel increases form stability and correlates with better shape retention when compared with saline or fluid form silicone filled implants [6]. 

Textured or smooth outer surface:

Smooth breast implants move within the breast implant pocket to give a more natural movement [5], while aggressive texturisation of the implant surface improves integration between the living host and the implant by enhancing tissue adhesion, growth and proliferation of the host blood supply, enhancement of cellular migration, and fibroblast adhesion [7,8]. Texturisation is thought to increase device stability as it helps prevent rotation in the breast pocket or migration of implants [5]. 

**Figure 1:**
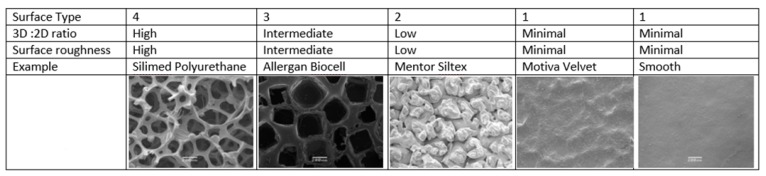
Implant surface classification and representative examples of implants.

Currently available breast implants can be categorised into 4 different surface types based on the 3D to 2D surface area ratio (high >5, intermediate 3–5, low 2–3 and minimal <2) and surface roughness expressed as a multiple of the value of smooth implants (high > 150, intermediate 75–150, low 25–75 and minimal <25) [9]. Figure 1 describes the implant surface classification system and representative scanning electron microscope pictures of breast implants.

The first textured implant, released in 1968, incorporated a 1.2–2 mm polyurethane foam (PU) coating on its outer surface, which adhered to the surrounding tissues, and subsequently delaminated from the silicon implant producing a relatively non-contractible capsule and thus reduced the risk of capsular contracture [10,11]. However, polyurethane (PU) coated silicone implants were voluntarily removed from the USA market in 1991, due to reporting of an association between polyurethane and the carcinogen 2,4-toluenediamine (TDA) [12]. This withdrawal lead to the development of alternative technologies to modify the outer silicone shell, including bonding the PU foam coating to the silicon surface, e.g., the Silimed PU implant, which retains the aggressive texture but prevents delamination [4]. This implant has been classified as surface type 4 (Figure 1) [9].

The salt-loss technique of producing a textured surface is produced by adding salt crystals to the silicone before curing, which are then washed from the surface leaving behind a pitted surface with randomly sized and arranged interconnected pores [13]. The pores promote adherence to the surrounding tissue [14,15,16] and make these devices relatively immobile [16]. Allergan Biocell is produced by the salt-loss technique and has pores with an average diameter of 600–800 μm and depth of 150–200 μm [15] and is an example of a surface type 3 implant (Figure 1) [9]. A micro textured implant (with an average pore size of 100 to 150 μm diameter) manufactured by Polytech Mesmo through a vulcanisation process that coats the surface of the uncured implant with ammonium carbonate [6,17] has also been classified as a surface type 3. 

Negative contact imprinting, such as with Mentor Siltex, creates a less aggressive textured silicone surface by pressing the uncured silicone mandrel into PU foam. This results in an implant surface of type 2 with average pore diameters of 70–150 μm and depth of 60–275 μm and is meant to mimic the PU foam (Figure 1) [15]. In contrast to Silimed PU and Biocell, Siltex does not adhere to the surrounding tissue and is not immobile [10]. Motiva, using a propriety method of negative imprinting, manufacture the nanotextured SilkSurface and the micro-textured VelvetSurface (Figure 1). The pore depth on the VelvetSurface is 40–100 µm [18] which is shallower than Mentor Siltex. Along with smooth implants, nanotextured implants are classified as surface type 1 (Figure 1) [9].

## 3. Capsular Contracture 

Complications of breast augmentation include hematoma, seroma, infection, altered nipple sensation, asymmetry, scarring, swelling, rupture, leakage and capsular contracture (CC) but CC is thought to be the most common complication and frequently requires surgical revision [19]. In 2015, 43,000 implant removal procedures were reported in the United States of America [1], and the Food and Drug Administration (FDA) reports that between 20 to 40% of augmentation patients and 40 to 70% of reconstruction patients had reoperations during the first eight to ten years after receiving their breast implants [20]. CC is a common reason for reoperation in Australia, being responsible for 38.9% of the 5290 breast implant revisions occurring between 2012 and 2016 [21]. Surgical revisions following CC result in poorer aesthetic outcome and a high rate of recurrence of CC [22,23]. 

Upon insertion of a breast implant, a foreign body reaction is induced, which is essentially an excessive fibrotic response that encloses the implant. CC is contracture of the peri-prosthetic capsule, which is characterised by the tightening and hardening of the tissue capsule around the breast implant. CC eventually leads to distortion of the implant [24,25]. Individual studies have reported incidence rates of CC ranging from 1.3 to 45% [23,26,27,28,29,30]. The wide range of CC rates is attributed to differences in follow-up times, as CC rate increases with time following implantation, as well as different type of implants and differing surgical techniques being used throughout the various studies [4]. 

The degree of CC is classified using the Baker clinical grading system which divides CC into four grades [31]. A grade I breast looks and feels natural, while grade II breasts have minimal contracture where the surgeon can tell surgery has been performed but there are no clinical symptoms. Grade III and IV are clinically significant and symptomatic, where grade III describes moderate contracture with some firmness felt by the patient, and grade IV describes severe contracture that is obvious from observation and symptomatic in the patient [31]. 

With each new implant generation, the incidence of CC has decreased, although whether this is due to implant design or better surgical technique, or a combination of both, is unclear. Historically, the type of fill was thought to influence the development of CC. Older generation silicone gel devices were characterised by higher gel bleeds and rupture rates compared to current generation implants [5,32,33]. The rates of CC were six-fold higher with these older devices than with devices containing low-bleed silicone gel fillings [34] or cohesive silicone gel fill implants [22,35,36,37,38,39].

The benefits of textured implants in reducing CC remains controversial. Systematic reviews of comparative clinical studies concluded texturisation may reduce the incidence of early capsular contracture if the implant was placed under the breast glandular tissue, but had no significant effect if placed under the pectoral muscle [29,40]. Smaller comparative or split breast studies, inserting one smooth and one textured implant in the same patient, are evenly divided as to the benefit of texturisation [41,42,43,44,45,46,47,48]. Many of these published reports lack adequate description of implant type, surgical technique, outcome assessment, and have short follow-up or the time period of follow-up is not stated. Several early randomised controlled trials reported textured implants had lower rates of clinically significant CC compared to smooth surface implants [42,45]. Similarly, some later prospective trials and metanalysis of 16 randomised controlled trials combined with two case-control studies, involving 4412 patients, have shown that smooth implants are more likely to develop CC [40,48,49]. However, the follow-up of most of these studies has been less than five years. When 715 of these patients were followed for 10 years there was no difference in the rate of CC [23]. It is likely that the effect of surface technology is of some benefit but is one of many factors that impact on clinical outcome, and the aetiopathogenesis of CC is likely to be multifactorial.

## 4. Aetiopathogenesis of Capsular Contracture (CC)

In 1981, Burkhardt and co-workers [50] were the first to propose that subclinical infection led to CC. However, the lack of culture positivity in many clinical studies of CC delayed the acceptance of this hypothesis. The detection of a *Staphylococcus epidermidis* biofilm in a patient with recurrent CC led to the hypothesis that the proposed subclinical infection is due to biofilm formation on the breast implant [51]. The presence of biofilm on implants obtained from CC patients was confirmed using scanning electron microscopy [25]. The likelihood of bacterial isolation was increased by mincing, sonication, and broth culture of a piece of implant or tissue, rather than using a swab to collect samples. Using this improved method of culture, the authors found a significant relationship between culture positivity (*p* < 0.0006) and the presence of *S. epidermidis* (*p* < 0.01) with CC. Subsequently, the degree of Baker grade CC has been shown to directly correlate with the number of bacteria in humans [52] and in the porcine model [53]. The biofilm hypothesis helps explain the lack of culture positivity in older studies where sonication was not employed, as biofilm bacteria are notoriously difficult to culture [54].

An alternative, to the biofilm hypothesis is that CC is purely an immunological response (reviewed in Headon [4]). The principal cell type within the capsule include activated macrophages, lymphocytes, and fibrocytes, and the number of lymphocytes and fibrocytes correlate with Baker grade [4]. However, the trigger for activating these cells is unknown. The presence of silicon particles has been postulated as a trigger. The amount of silicon in capsular macrophages is greater in higher grade CC and is associated with increased inflammation [55]. In contrast, the biofilm hypothesis proposes that the immunological response is activated by biofilm infection. The patient’s endogenous flora or bacteria present at the time of surgery gain access to the breast implant during or following placement. Once in contact with the implant, they attach to the prosthetic surface and form a biofilm. If implants are contaminated with only low numbers of bacteria, the host can contain the biofilm to a level that produces minimal inflammation [53]. However, once bacterial numbers reach a critical point, the host response is overwhelmed, and the bacteria continue to proliferate and trigger a chronic inflammatory response, leading to subsequent fibrosis and accelerated CC [53]. 

Frequently, organisms that are part of the microflora of the skin or the breast, such as *Cutibacterium acnes* (formally *Proprionibacterium acnes*) and coagulase-negative staphylococci, particularly *S. epidermidis*, are commonly isolated from CC samples [25,50,52,56,57,58,59]; however, any bacterial species can be involved and multiple species can be grown from one breast [25,59]. 

Further evidence for bacterial involvement in CC aetiopathogenesis is provided by artificial inoculation of implants, resulting in increased CC development in animal models [56,60,61]. In the porcine model, breast pocket inoculation of *S. epidermidis* led to biofilm development, and biofilm formation was associated with a four-fold increased risk of developing contracture (odds ratio = 4.1667) [61]. 

Additional evidence to support the subclinical biofilm hypothesis is that strategies to prevent breast implant infection appear to be effective. Animal studies have shown that antimicrobial coated implants can significantly reduce the genesis of biofilm and subsequent CC [62,63], whilst clinical studies utilising antibiotic or antiseptic breast implant pocket irrigation at time of surgery have shown a significant reduction in CC [24,64]. The reduction in CC following biocide irrigation has been confirmed in two comparative clinical trials that showed a 10-fold reduction in CC utilising either betadine and/or topical antibiotics in pocket irrigation [65,66].

Other strategies to prevent bacterial contamination of the implant by modifying surgical technique have resulted in decreased CC rates (reviewed by Deva et al. [19]). These include modification of implantation site (subpectoral position reduces access of breast flora to the implant through the natural musculofascial barrier); avoiding periareolar and transaxillary incisions, which have higher rates of CC compared to submammary incisions; use of a nipple shield; and use of an introductory shield to prevent the implant touching the skin surface [19]. 

The occurrence of unilateral contracture following bilateral insertion of identical breast implants means that systemic or implant material-related causes are also less likely [52]. Thus, although contracture remains poorly understood, it is likely to be multifactorial in origin, and of all the theories on the potential aetiology of CC, the subclinical infection hypothesis remains the leading theory.

## 5. Breast Implant Associated Anaplastic Large Cell Lymphoma

In 2011, the FDA identified a possible association between textured breast implants and anaplastic large cell lymphoma (BIA-ALCL) [67], a rare T- or null-cell non-Hodgkin lymphoma first described by Stein and co-workers [68]. It was recognised as a distinct cancer by the World Health Organisation in 2016 [20]. As of 2017, over 500 cases were reported worldwide, and recent epidemiological studies suggest that the number will continue to rise [69,70,71]. Australia has a high incidence rate with 70 confirmed cases of BIA-ALCL, including four deaths by August 2016 [70,72]. The Australian Therapeutic Goods Administration estimates the risk of developing BIA-ALCL to be between 1:1000 and 1:10,000 for women with breast implants [70,72]. However, the true incidence of BIA-ALCL is likely to be higher due to under reporting and the lack of accurate breast implant sales figures.

BIA-ALCL generally presents as a localised late peri-implant seroma containing malignant cells in one breast and less commonly as a tumour mass attached to the capsule, and regional lymph node involvement is seen in around 5–10% of patients. In the Australian cohort, all patients were exposed to textured implants with 85% of cases associated with implants with a high surface area (surface type 3 or 4, Figure 1) [70]. BIA-ALCL occurs an average of seven to ten years after implant placement but can range from 0.4 to 20 years [70,73,74,75]. Treatment for the majority of patients consists of complete surgical excision of diseased tissue, implants, and the surrounding fibrosis capsule, while adjuvant chemotherapy is only recommended for patients with advanced disease (reviewed by Clemens and co-workers [76]).

BIA-ALCL seroma fluid is composed of large, pleomorphic cells with horseshoe-shaped nuclei and are anaplastic lymphoma kinase (ALK) negative. Immunophenotypically they are diffusely positive for CD30 and T-cell markers such as CD3, CD4 [76,77,78,79,80]. Additionally, in cell lines developed from clinical cases of BIA-ALCL antigen presentation markers (HLA-DR, CD80, CD86), IL-2 receptors (CD25, CD122) and IL-6 receptors are present [80,81,82]. BIA-ALCL cells show clonal TCR gene arrangement and/or the demonstration of phenotypic aberrancy, including CD4 and CD8 co-expression [76,79,80]. 

The aetiopathogenesis of BIA-ALCL is unknown, but it is thought that chronic inflammatory stimulus leads to T-cell dysplasia in patients that are genetically susceptible. It is postulated that a milieu rich in immune stimulatory cytokines, which promotes rapid division of host lymphocytes, may cause the initial tumorigenic changes that lead to BIA-ALCL in some patients. Autocrine production of IL-6 has been identified as a driver of tumorigenesis in some diffuse large B-cell lymphomas, as well as solid tumours, including breast, lung, and ovarian carcinomas [83,84,85]. The cytokine profile of BIA-ALCL cell lines, specifically IL-6, TGF-β and IL-10, has also been shown to induce immune suppressor cell populations (Tregs and myeloid-derived suppressor cells), which may inhibit host anti-tumour immunity and facilitate cancer development [86,87]. 

**Figure 2:**
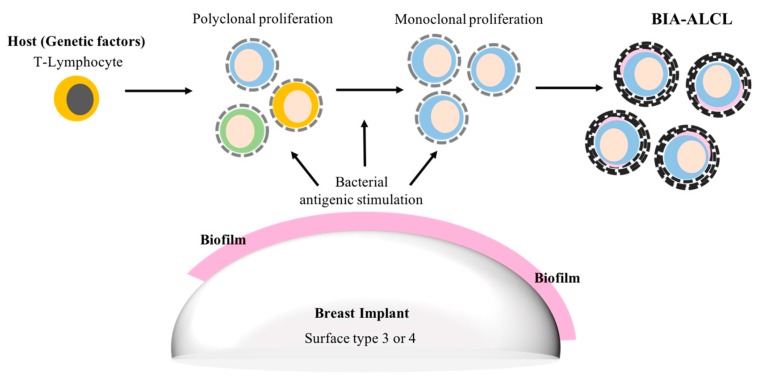
Suggested biofilm aetiopathogenesis of breast implant associated-anaplastic large cell lymphoma (BIA-ALCL).

One theory is that biofilm infection, combined with host factors such as the patient’s genetic background and their immune response, activate T-lymphocytes and trigger polyclonal proliferation and, with time, in some cases monoclonal proliferation and the eventual development of ALCL [88] Figure 2. 

In support of the biofilm infection theory, chronic biofilm infection with *Helicobacter pylori*, and hence ongoing inflammation, is recognized as being the causal agent in the development of gastric lymphoma [89], and antibiotic treatment alone in patients with low grade malignancy results in remission in 80% [90]. Similarly, a phase II clinical trial showed regression of adnexal marginal zone lymphoma in 65% of patients given doxycycline monotherapy for the treatment of *Chlamydophila psittaci* (*n* = 34) [91]. Therefore, it is plausible that chronically infected breast implants may mediate similar inflammatory and neoplastic processes resulting in the development of a T cell lymphoma. In support of biofilm being the chronic inflammatory stimulus, significantly more bacteria attach to textured implants compared to smooth implants [9]. In the porcine model this correlated with a 63-fold increase in the number of lymphocytes attached to textured implants compared to smooth implants, whilst in clinical samples of CC the number of lymphocytes surrounding breast implants is positively and significantly correlated (*r* = 0.83) with the number of bacteria [53].

**Figure 3:**
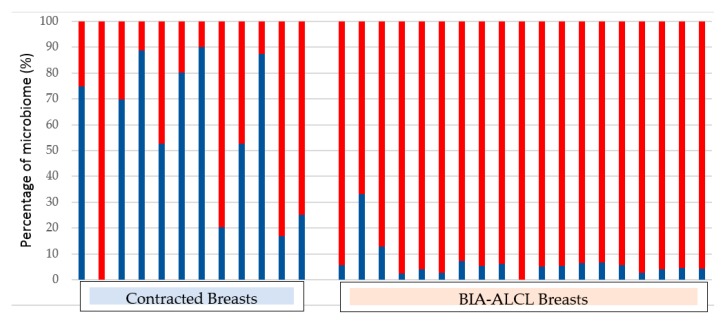
Percentage of Gram-positive (coloured blue) and Gram-negative (coloured red) organisms in capsules obtained from contracted breasts and in BIA-ALCL samples [88].

The strongest support for the role of bacterial biofilm in the aetiopathogenesis of BIA-ALCL was the detection of biofilm in clinical samples using qPCR, with visual confirmation of biofilm presence using fluorescent in situ hybridisation and scanning electron microscopy [88]. Analysis of the microbiome (bacterial community genetic profile), using next generation sequencing, showed a significantly greater proportion of Gram-negative bacteria in BIA-ALCL specimens compared with non-tumour CC specimens (Figure 3), suggesting that different bacterial species may preferentially trigger lymphocyte activation [88].

The development of BIA-ALCL is likely to be a complex process resulting from an interplay of host, implant and microbial factors, including the patient’s genetic background, immune response, the textured implant surface, and bacterial phenotype. However, the rarity of BIA-ALCL presents a challenge for conducting meaningful epidemiologic studies, and although the pathogenesis of BIA-ALCL is undergoing active research, the drivers of this malignancy remains poorly understood.
